# Peptides OFFGEL electrophoresis: a suitable pre-analytical step for complex eukaryotic samples fractionation compatible with quantitative iTRAQ labeling

**DOI:** 10.1186/1477-5956-6-9

**Published:** 2008-02-26

**Authors:** Jérôme Chenau, Sylvie Michelland, Jonathan Sidibe, Michel Seve

**Affiliations:** 1 Centre de Recherche INSERM/UJF U823, Cibles Diagnostiques ou Thérapeutiques et Vectorisation des Drogues dans le Cancer du Poumon Institut Albert Bonniot, 38706 La Tronche, France; 2 Université Joseph Fourier, 38041 Grenoble Cedex 09, France; 3 Centre d'Innovation en Biologie, Pavillon B, CHU de la Tronche, 38043 Grenoble Cedex 9, France

## Abstract

**Background:**

The proteomes of mammalian biological fluids, cells and tissues are complex and composed of proteins with a wide dynamic range. The effective way to overcome the complexity of these proteomes is to combine several fractionation steps. OFFGEL fractionation, recently developed by Agilent Technologies, provides the ability to pre-fractionate peptides into discrete liquid fractions and demonstrated high efficiency and repeatability necessary for the analysis of such complex proteomes.

**Results:**

We evaluated OFFGEL fractionator technology to separate peptides from two complex proteomes, human secretome and human plasma, using a 24-wells device encompassing the pH range 3–10. In combination with reverse phase liquid chromatography, peptides from these two samples were separated and identified by MALDI TOF-TOF. The repartition profiles of the peptides in the different fractions were analyzed and explained by their content in charged amino acids using an algorithmic model based on the possible combinations of amino acids. We also demonstrated for the first time the compatibility of OFFGEL separation technology with the quantitative proteomic labeling technique iTRAQ allowing inclusion of this technique in complex samples comparative proteomic workflow.

**Conclusion:**

The reported data showed that OFFGEL system provides a highly valuable tool to fractionate peptides from complex eukaryotic proteomes (plasma and secretome) and is compatible with iTRAQ labeling quantitative studies. We therefore consider peptides OFFGEL fractionation as an effective addition to our strategy and an important system for quantitative proteomics studies.

## Background

The proteomes of mammalian cells, tissues and body fluids are complex and display a wide dynamic range of proteins concentration. In order to overcome the human proteome complexity and determine the proteome content, it is necessary to use sample fractionation steps.

The recent introduction of commercially available OFFGEL fractionator system by Agilent Technologies, provides an efficient and reproducible separation technique [1,2]. This separation is based on immobilized pH gradient (IPG) strips and permits to separate peptides and proteins according to their isoelectric point (pI), but is realized in solution [1,3]. Therefore, its micropreparative scale provides fraction volumes large enough to perform subsequent analyses as reverse phase (RP) – liquid chromatography (LC) – MALDI MS/MS.

The secretome, first introduced by Tjalsma *et al *in 2000, describes the global study of secreted proteins by a cell, tissue or organism at any given time or under certain conditions [4,5]. Secretome is the origin of circulating proteins in the body and a very promising source for discovery of new biomarkers candidates. Human plasma is the most complex body fluid and contains a large number of proteins with a dynamic range of at least 9–10 orders of magnitude [6]. This complexity is a problem for proteomic analysis and it is necessary to develop efficient separation techniques to determine its precise protein composition.

In this study, we applied OFFGEL fractionation in combination with RP LC-MALDI MS/MS analysis to separate peptides of complex samples as secretome and plasma and we analyzed and explained the specific repartition profiles obtained. We also evaluated the efficiency of OFFGEL fractionation with peptides previously labeled by the isobaric amine-specific tags used in recently developed iTRAQ™ (isobaric tags for relative and absolute quantification) technology [7]. The validation of OFFGEL electrophoresis on separation of eukaryote tryptic digests will permit to include it in proteomic workflow of complex samples.

## Results and discussion

**Figure 1:**
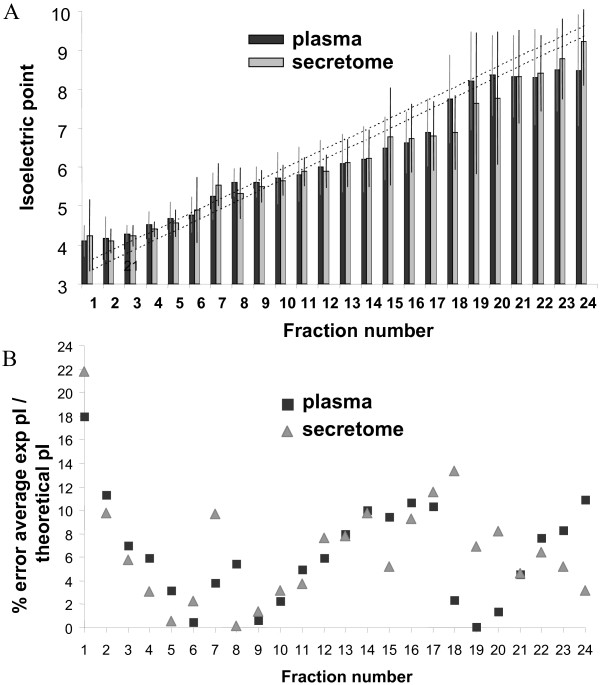
**Comparison of experimental and theoretical pI for each OFFGEL fraction**. (A) **Average experimental pI values for all peptides identified per fraction**. The dark area relates to the plasma peptides and the white area to the secretome peptides. Standard deviation is represented by errors bars. The broken lines are based on the theoretical pI values calculated according to the supplier's specifications of the IPG gel strip of 24 cm length (pH 3–10). (B) **Percentage error between the average experimental pI and the average theoretical pI per fraction.** Black squares correspond to the error for plasma and triangles correspond to the error for secretome.

**Figure 2:**
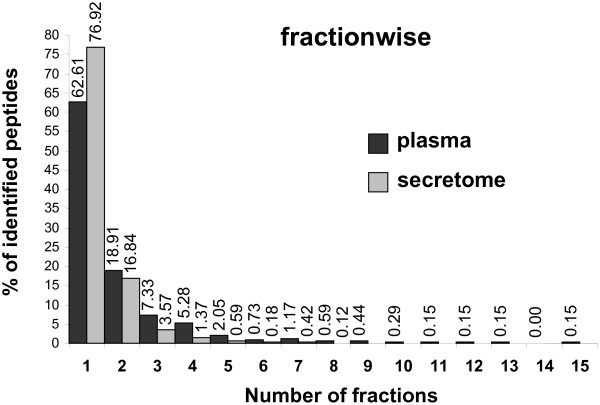
Fractionwise distribution of identified peptides in plasma sample (grey bars) and secretome sample (white bars).

The OFFGEL fractionator system was used in order to separate secretome and depleted plasma peptides according to their pI using a 24-wells device encompassing the pH range 3–10. The peptides are recovered in liquid phase, which is much more convenient for the others subsequent separation experiments like liquid chromatography. Expected pH ranges by fraction were calculated according to the IPG strip supplier data and the fractions size (Table 1). The attempted resolution was at least 0.3 pH units [1]. The average experimental pI for each fraction was computed from the peptides lists after filtering for false positives and replicates (Figure 1A and Table 1). Experimental pI values were globally similar for both secretome and plasma samples. The average experimental pI deviated from the average theoretical pI values with an average error of 6.36% for plasma and 6.69% for secretome. Error was < 6% in fractions 3–11 (pH 4.0–6.1) and was > 10% in fractions 14–17 (pH 6.8–7.7) (Figure 1B). The observed error increase was correlated with the low number of peptides found in this pH range and previously reported by Horth *et al *[2]. Standard deviations were weak for fractions 1–9 (pH 3.5–5.5) and relatively high for fractions 18–24 (pH 7.81–9.65). Globally, standard deviation increased from the neutral pH region to the basic pH region. This increase is correlated with the focalization level assessed by the ratio of unique peptides/total peptides, smaller for fractions in neutral and basic pH regions. Indeed, the most unique peptides to each fraction were found in fractions 2–5 (pH 3.6–4.7). This is in agreement with previously published studies reported that unique peptides to each fraction are more found in acidic region rather than neutral and basic regions [2,8-10]. The OFFGEL good quality separation can be validated looking at the number of unique peptides, identified only in a single fraction. 62.6% and 76.9% of peptides were found in a single fraction in plasma and secretome samples respectively (Figure 2). We observed that 81.5% and 93.7% of peptides were found in one or two fractions in plasma and secretome samples respectively. This result correlates with the findings of Hörth et al, who found that 74% of tryptic peptides of *E. Coli *focus in one well and 90% focus in two wells [2]. On the other hand, the worst separated peptide is found in 15 distinct fractions for plasma sample and in 8 distinct fractions for secretome sample. Although plasma sample has been depleted before OFFGEL fractionation, the sample remained complex because of the large dynamic range of protein concentration and the difficulty to remove the totality of the higher abundant proteins.

**Table 1:** The expected pH ranges per fraction were calculated according to the IPG strip supplier data and the OFFGEL well dimensions and compared with experimental pI obtained for plasma and secretome samples.

			**Plasma**		**Secretome**	
**Fraction**	**Expected pH range**		**Peptide pI Average**	**s.d.**	**Peptide pI Average**	**s.d**

1	3.35	3.61	4.11	0.40	4.24	0.92
2	3.61	3.88	4.17	0.56	4.11	0.31
3	3.88	4.14	4.29	0.22	4.24	0.26
4	4.14	4.40	4.53	0.32	4.40	0.19
5	4.40	4.66	4.67	0.42	4.56	0.33
6	4.66	4.93	4.77	0.45	4.90	0.84
7	4.93	5.19	5.25	0.59	5.55	0.54
8	5.19	5.45	5.61	0.36	5.33	0.65
9	5.45	5.71	5.61	0.39	5.50	0.41
10	5.71	5.98	5.71	0.67	5.66	0.39
11	5.98	6.24	5.81	0.70	5.88	0.36
12	6.24	6.50	5.99	0.70	5.88	0.42
13	6.50	6.76	6.10	0.75	6.11	0.61
14	6.76	7.03	6.20	0.85	6.22	0.72
15	7.03	7.29	6.48	0.80	6.78	1.24
16	7.29	7.55	6.63	0.78	6.73	0.87
17	7.55	7.81	6.89	0.87	6.79	0.88
18	7.81	8.08	7.75	1.13	6.88	0.95
19	8.08	8.34	8.20	1.26	7.64	1.81
20	8.34	8.60	8.36	1.03	7.78	1.70
21	8.60	8.86	8.33	1.05	8.33	1.20
22	8.86	9.13	8.31	1.23	8.42	0.97
23	9.13	9.39	8.49	1.06	8.77	1.04
24	9.39	9.65	8.48	1.42	9.22	1.13

(s.d. = standard deviation)

**Figure 3:**
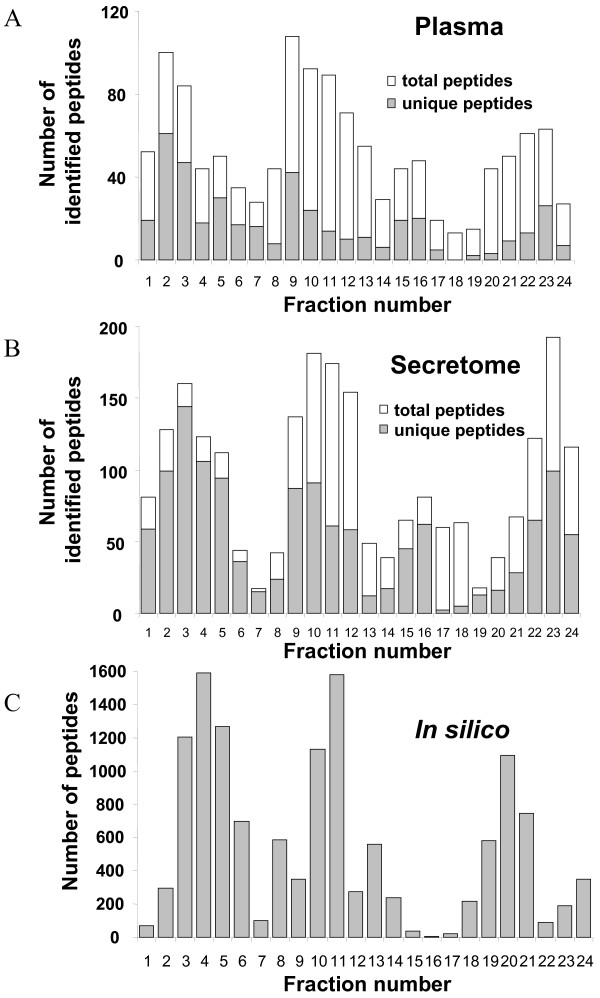
**Total number of peptides identified per fraction: (A) **in plasma sample and **(B) **in secretome sample. The grey area relates to the unique peptides in each fraction. **(C) **I*n silico *repartition of all the peptides identified for the 9504 plasma proteins from the Human Plasma Project found on the HUPO website.

**Figure 4:**
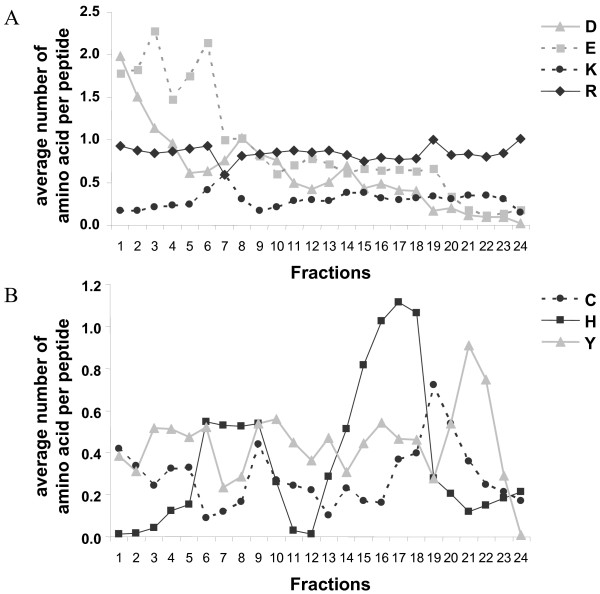
**Average distribution of charged amino acids per peptide in each OFFGEL fraction obtained for secretome separation**. **(A) **Average number of acidic amino acids (aspartic acid (D) and glutamic acid (E)), and basic amino acids (lysine (K) and arginine (R)).**(B) **Average number of cysteine (C), histidine (H) and tyrosine (Y) charged amino acids.

Peptides were unevenly distributed along the IPG strip scale (Figure 3A and 3B). Gaps were observed in fractions 6–7 (pH 4.66–5.19), 14 (pH 6.76–7.03) and 17–19 (pH 7.55–8.34). To more understand the factors determining peptides pI distribution, we digested *in silico *the 9504 plasma proteins identified from the Human Plasma Project [11] and calculated the pI distribution of the 14800 resulting peptides, after elimination of replicate peptides. Interestingly, graphical representation of the pI distribution of these peptides fits fairly with those obtained from our experimental data (Figure 3C). Gaps were found at fractions 7 and 15–17. For gaps in neutral and basic region, *in silico *distribution is slightly shifted on the left and could be explained by trypsin miss-cleavages in experimental data. These results correspond to Hörth's study using OFFGEL fractionation of total *E.Coli *peptides [2] and to theoretical results of Lam *et al *[12]. So the repartition across the OFFGEL IPG strip of proteome tryptic digest is not dependent on the sample nature or organism (eukaryote or prokaryote). Some molecular considerations have been introduced to explain the unevenly distribution of peptides. We calculated the average number of each amino acid by peptide and by fraction. We then focused on the 7 charged amino acids aspartate (D), glutamate (E), arginine (R), lysine (K), histidine (H), tyrosine (Y), cysteine (C) which mainly contribute to the overall charge of the peptides and used for the theoretical calculation of their pI [13]. As expected, the number of acidic amino acids D and E by peptide globally decreases from acidic to basic fractions (Figure 4A). Acidic peptides (Fraction 1–3) have an average of 2 D or E amino acids while basic peptides have less than 0.2 acidic amino acid. The number of the basic amino acids R and K is globally constant in each fraction, explained by the action of trypsin, cutting proteins after these residues. Expecting no miss-cleavage, each peptide should contain an arginine or a lysine residue at the c-terminus. Arginine is more frequently observed: 80% versus 20% of the peptides containing a lysine residue. In our experimental conditions, cysteine residues do not contribute to the charge of the peptides as they were reduced and alkylated prior the OFFGEL separation. However, cysteine repartition is not homogenous along the strip with less cysteine in the peptides migrating in fractions 6 to 8, which contain a weak number of peptides (Figure 4B). Conversely, fractions 19 and 20 which are also low abundant fractions have peptides with a high content in cysteine residues. Tyrosine is present in all fractions but is more abundant in the basic fractions because of its basic side-chain. Histidine is present mainly in two regions: fractions 6–9 and 15–18. It is also important to note that the mean size of the peptides decreases from 15 amino acids in acidic fractions to 10 amino acids in basic fractions.

**Figure 5:**
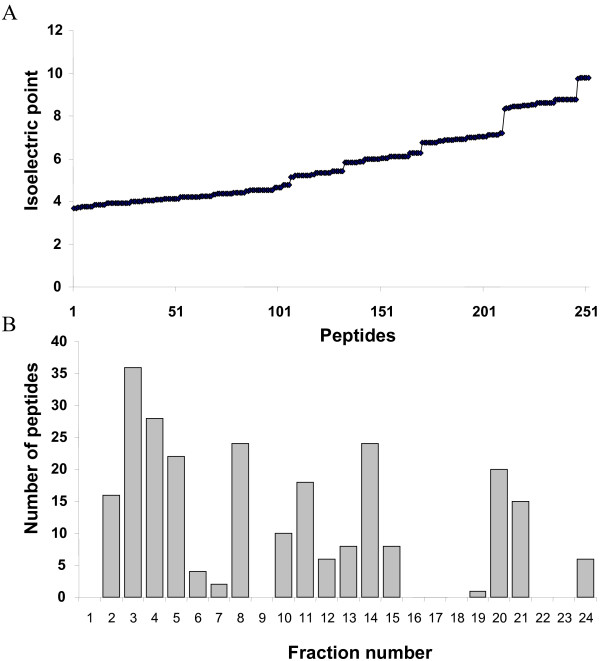
**Analysis of peptides generated by a calculated model**. Our model is based on the generation of all possible peptides containing a combination of 6 amino acids out of the 6 charged amino acids contributing to the pI calculation (D, E, R, K, H, Y). Only one arginine or lysine is theoretically present in a given peptide after tryptic digestion. **(A) Isoelectric point repartition of peptides obtained by the combination model**. Generated peptides are classified by increasing theoretical pI. **(B) Distribution of theoretical peptides per fraction. **Generated peptides are classified in function of their theoretical pI correspondence to the pH range of each fraction.

We built a calculated model of the OFFGEL peptide separation based on the generation of all possible peptides containing a combination of 6 amino acids out of the 6 charged amino acids contributing to the pI calculation (D, E, R, K, H, Y) with the condition that only one arginine or lysine is present in a given peptide. 96% of the peptides sequenced from plasma or secretome have 0 to 6 charged amino acids. We generated 252 theoretical combinations and calculated their respective pI. We obtained a set of possible pI with a profile globally homogenous up to the 7^th ^fraction and the presence of steps in the pI scale after this fraction (Figure 5A). These steps explain the higher standard deviation obtained for the basic fractions. From this set of pI, peptides were reattributed to the different fractions (Figure 5B). It does not exist any combination resulting in a peptide with a pI below 3.66. That explains why the average pI observed for the first fraction is higher than expected by the strip specifications and why the first fraction standard deviation is so high. The same problem was observed for fractions 16 to 18. In our model, it does not exist any combination of charged amino acids resulting in a peptide with a pI in the range 7.29 to 8.08 corresponding to these fractions. Last, no combination generated a pI over 9.78. However, we observed experimentally a lot of peptides with pI between 10 and 14, accumulating in the fraction 24. The analysis of their sequence revealed miss-cleavages with two adjacent lysine or arginine residues. By comparison of the profile obtained from the model and experimental data (Figure 3), we observed that the fractions with few peptides, *i.e*. fractions 6 and 7 (pH 4.66–5.19) and fractions 16 and 19 (pH 7.29–8.34) correspond with a lack of possible combinations of charged amino acids resulting in peptides in these ranges of pH (Figure 5B). A previous study has shown the compatibility of the recently developed isotopic labeling technique iTRAQ with isoelectrofocalisation separation (in-gel) [14]. We wanted here to study the compatibility of OFFGEL fractionation with iTRAQ. We labeled the secretome sample with iTRAQ reagent and separated the peptides with OFFGEL following the same protocol as the unlabelled sample for the separation. For this purpose, the peptide composition of three different fractions in different pH range was compared (fractions 2: acidic pH; 11: neutral pH and 24: basic pH) (Table 2). iTRAQ labeling did not affect the separation for the fractions 2 and 11, as the mean pI was identical for these fractions with or without labeling and a majority of peptides were found present in the same fraction in both conditions (68.1 % and 74.1 % respectively). For fraction 24, we observed a slight modification of the pI by iTRAQ labeling (ΔpI = 0.48) and a lower percentage of coverage. It could be explained by a higher number of miss-cleaved peptides recovered from this fraction. To conclude, we showed for the first time the compatibility of iTRAQ labeling with OFFGEL separation. OFFGEL fractionation is particularly interesting for quantitative proteomic analysis because higher amounts of sample can be loaded and separated, allowing an optimization of the subsequent analytical steps. These results were also validated for plasma samples (data not shown), a complex sample that may require the coupling of two or three separation stages in order to access to the less abundant proteins. For example, a combination of OFFGEL, Strong Cation Exchange (SCX) and nano RP chromatographies seems to be an interesting approach to apprehend the complexity of the plasma. An another advantage of OFFGEL is its ability to desalt the sample during separation which can avoid in certain processes a supernumerary desalting step [15].

**Table 2:** iTRAQ labeling influence on peptides repartition for OFFGEL fractions 2, 11 and 24 for secretome sample.

	**+ iTRAQ**		**- iTRAQ**			
**Fraction**	**Peptide pI Average**	**s.d.**	**Peptide pI Average**	**s.d.**	**ΔpI**	**% of Coverage**

**2**	4.21	0.66	4.11	0.31	0.10	68.12
**11**	5.88	0.53	5.88	0.36	0.00	74.14
**24**	8.74	1.35	9.22	1.13	-0.48	56.90

(s.d. = standard deviation; ΔpI = difference of the peptide pI average for a same fraction with and without iTRAQ labeling)

## Conclusion

Sample fractionation is an essential step for proteomics analysis. The data reported here show that OFFGEL system provides a highly valuable tool to fractionate peptides from complex eukaryotic samples like plasma or secretome. We also demonstrated the compatibility of OFFGEL fractionation with the iTRAQ labeling reagents used for quantitative proteomics. Finally, we consider peptides OFFGEL separation as a highly valuable technique to integrate in a proteomic workflow for complex sample analysis.

## Methods

### Plasma samples

For quality and standardization issues, the plasma samples used were IQC samples (Internal Quality Control) provided by the proteomic laboratory of Dijon (Plateform Proteomic IFR- santé-STIC, France) as calibrated and reference samples. IQC was a pool of inactivate plasma packaged in controlled conditions. The samples were received in 50 μL aliquots and stored at -130°C until use. The samples were depleted using PROTEOPREP 20S spin column technology, according to the procedure recommended by Sigma-Aldrich, which remove the 20 highest abundant proteins. After 10 depletion cycles, corresponding to 80 μL of crude plasma, depleted solutions are recovered and then concentrated by ultra filtration using a 5 kDa molecular mass cut-off spin column (Amicon Ultra, Millipore).

### Secretome samples

H358 human non-small lung adenocarcinoma cell line [16] were grown in RPMI-1640 medium with glutamine, 10% heat-inactivated fetal bovine serum and Penicillin/Streptomycin 5000 U in 175-cm^2 ^dishes until they reached a confluence state of approximately 60–70 %. They were then gently washed four times with phosphate buffered saline and two times with serum free medium to eliminate serum contaminants and left in serum free medium for 72 hours. The conditioned medium was collected and cooled down on ice. Floating cells and cellular debris were removed by centrifugation (200 × g, 10 min) followed by sterile filtration (pore size: 0.2 μm). Proteins were then concentrated and desalted by ultra-filtration using a 5 kDa molecular mass cut-off spin column (Amicon Ultra, Millipore) according to the manufacturer instructions. The total protein amount was determined using a standard Bradford protein assay (Bio-Rad).

### Tryptic digestion

Secretome and plasma samples were treated simultaneously and with the same protocol. 200 μg of each sample were reduced with 45 mM dithiothreitol at 50°C for 35 min and alkylated with 100 mM iodoacetamide at room temperature for 45 min. Trypsin (Promega) was added at an enzyme: protein ratio of 3:100 w/w and incubated overnight at 37°C. The digests were dried by vacuum centrifugation prior to the OFFGEL peptides fractionation.

### iTRAQ labelling

200 μg of proteins from secretome sample were resuspended, reduced, alkylated, and digested according to the standard protocol supplied by the manufacturer (Applied Biosystems). Then, 100 μg of each digest were labeled either with iTRAQ reagent 114 or iTRAQ reagent 117. After labeling, samples were pooled in a ratio 1:1 (v/v) (for a total of 200 μg of peptide digests) and dried by vacuum centrifugation prior to the OFFGEL peptides fractionation.

### OFFGEL peptides fractionation

To perform peptide fractionation according to their pI, the 3100 OFFGEL Fractionator and the OFFGEL Kit 3–10 (both from Agilent Technology) were used following the user protocol. The device was set up for the 24 fractions separation by using 24-cm-long IPG gel strip with a linear pH gradient ranging at 3–10. The peptides are separated in a two-phases system: liquid upper phase (focusing buffer provided by the supplier) separated in wells and lower IPG gel strip phase. The wells are isolated from each others. There is no direct fluidic connection between the wells. The peptides migrate through the IPG gel that plays the role of 'bridge" between each well and are retrieved in the solution at the IPG region where pH is peptides pI. 200 μg of secretome (with or without iTRAQ labelling) or plasma tryptic digests were resuspended with focusing buffer to a final volume of 3.6 mL. 150 μL of this sample was loaded in each of the 24 wells. The sample was focused using the recommended method for OFFGEL peptides 24 wells fractionation with a maximum current of 50 μA. The focusing was stopped after total voltage reaches 50 kVh. During the focusing, oil was added to the electrodes to prevent any evaporation effect. After focusing, 50 to 150 μl of sample was recovered for each well and transferred in individual micro tubes. To recover as much as possible the focusing peptides, 150 μl of methanol was added to each well, incubated for 15 min without voltage [3]. Corresponding peptides fractions were pooled and concentrated by vacuum centrifugation prior to LC-MALDI MS/MS analysis.

### Nano Reversed-phased LC-MALDI MS/MS analysis

Peptides were re-dissolved in 20 μl 0.2% trifluoroacetic acid. Peptides separation was performed on an Ultimate nanoHPLC System (Dionex/LC Packings, France) equipped with a PepMapC18 column (Dionex/LC Packings; 3-μm particles, 10 nm pore size, 75-μm *i.d*.), an autosampler and a Probot microfraction collector. The mobile phase consisted of a gradient of solvents A (0.05% trifluoroacetic acid; 2% acetonitrile in water) and B (0.05% trifluoroacetic acid; 80% acetonitrile in water). Injection was performed with 100% solvent A. The peptides were separated with a linear gradient of solvent B from 0–5% in 5 min, followed by an increase until to 40% of solvent B in 30 min and to 55% in 10 min at a flow rate of 0.3 μL/min. The column was washed and regenerated with 90% solvent B for 10 min and with 100% solvent A. For MALDI MS/MS analysis, column effluent was mixed in a 1:3 ratio with MALDI matrix (2 mg/mL α-cyano-4-hydroxycinnamic acid in 0.1% trifluoroacetic acid/70% acetonitrile) (v/v) and deposed on an Opti-tof LC/MALDI Insert 123 × 81 mm plate (Applied Biosystems) at a frequency of one spot/15s.

MALDI plates were analyzed by MALDI TOF TOF 4800 proteomics Analyzer mass spectrometer (Applied Biosystems) in positive reflector ion mode. MS spectra from m/z 700–3500 were acquired for each spot using 1500 laser shots. The ten most intense peaks in each MS spectrum above an S/N threshold of 100 were selected for MS/MS analysis.

### Data analysis

Peptides and proteins identification were performed using the GPS (Global Proteome Server) Explorer software V3.6 (Applied Biosystems) with Mascot (Matrix Science) as the database search engine (V2.0). Each MS/MS spectrum was searched against a database of human protein sequences (Swiss-Prot, downloaded January 2006), resulting in a set of tryptic peptides matches with confidence values. Only the peptides with C.I.% > 85%, for any MS/MS spectrum were retained for further analysis. These peptide identifications were then combined using the MASCOT search engine to yield a set of human protein identifications with confidence values. The MASCOT searches were run using the following parameters: methionine oxidation, cystein carbamidomethylation modifications were selected as variable; 1 missed cleavage allowed; precursor error tolerance at < 50 ppm; MS/MS fragment tolerance set to 0.2 Da and charge set to +1; full trypsin specificity (N- and C-terminal also applied). Only proteins with at least two specific peptides matched were considered positively identified.

The theoretical pI of the peptides, were calculated using "Compute pI/Mw" tool accessible on the Expasy website [13,17]. The identified peptides with an ion score C.I.% higher than 95% were always kept in the peptide list even if theoretical pI did not match with pI of the relevant individual OFFGEL fraction. For peptide ion score C.I.% between 85% and 95%, peptides were kept if calculated theoretical pI was correlated with pH range ± 2.

## Abbreviations

LC, Liquid Chromatography; pI, Isoelectric point; MALDI, Matrix Assisted Laser Desorption Ionization; MS, Mass Spectrometry; IPG, Isoelectric Point Gel; IEF, IsoElectric Focusing; IQC, Internal Quality Control; HUPO, Human Proteome Organisation.

## Competing interests

The author(s) declare that they have no competing interests.

## Authors' contributions

JC performed the laboratory experiments on secretome samples, carried out the LC separation of peptides samples and the MALDI-TOF/TOF analysis, and assisted in the writing of the final manuscript. JS performed the plasma samples depletion and participated in LC separation and MALDI-TOF/TOF analysis. SM participated in the design of the study and wrote the manuscript. MS supervised and coordinated the project. All authors read and approved the final manuscript.
